# Beyond emotional intelligence: The new construct of meta-emotional intelligence

**DOI:** 10.3389/fpsyg.2023.1096663

**Published:** 2023-04-05

**Authors:** Antonella D'Amico, Alessandro Geraci

**Affiliations:** Department of Psychology, Educational Science and Human Movement, University of Palermo, Palermo, Italy

**Keywords:** emotional intelligence, IE-ACCME test, ability EI, self-reported EI, perceived EI, meta-emotional intelligence, trait EI

## Abstract

Meta-emotional intelligence is a recently developed multidimensional construct that, starting from the original ability model of emotional intelligence, focuses on the cognitive aspects of emotional abilities and on the metacognitive and meta-emotional processes that influence our emotional life. Thus, meta-emotional intelligence is the combination of emotional abilities and meta-emotional dimensions, such as the beliefs about emotions, the self-concept about one's emotional abilities, and the self-evaluation of performance. This article aims to illustrate the theoretical and methodological background of this construct and to describe the IE-ACCME test, an original multi-method tool that has been developed to measure the different variables that compose meta-emotional intelligence. Applications of this construct will be discussed, as well as future directions.

## Introduction

Over the past 25 years, many authors dedicated their studies to the development of theoretical models and assessment tools of emotional intelligence (EI), proving that it is an important psychological dimension that influences various aspects of everyday life and health. However, both models and assessment tools of EI are extremely different from each other, and this produced a big and still active debate in the scientific literature [from the first reviews by Schulze and Roberts (2005), or Schutte et al. (2007) to the more recent paper by Fernández-Berrocal and Extremera (2016), Bucich and MacCann (2019), O'Connor et al. (2019), Bru-Luna et al. (2021). Mayer and Salovey (1997), in their four branches of the ability model, defined EI as a set of cognitive skills involved in perceiving, facilitating, understanding, and managing emotions. In 2016, Mayer, Caruso, and Salovey revisited their first model, defining emotional intelligence as a mental ability involved in processing “hot” information and positioning it among other hot intelligence such as personal and social intelligence. On the contrary, other theoretical proposals (Goleman, 1995; Bar-On, 1997, 2004; Petrides and Furnham, 2001; Petrides et al., 2007a) conceived EI as a combination of traits, competencies, and skills. These differences led to a well-known distinction among ability EI, trait EI, and mixed EI (Mayer et al., 2000, 2004; Petrides and Furnham, 2000) even if in more recent years, only the distinction between ability EI and trait EI is most often referred to (Petrides et al., 2016). In Petrides' perspective, trait EI ≪concerns people's perceptions of their emotional world≫ (Petrides et al., 2016, p. 335), and it is placed at the lower levels of personality hierarchies (Petrides et al., 2007b). Thus, trait EI is epistemologically described as a set of personality dimensions rather than as a form of intelligence, even if the authors of mixed and trait models defended the use of the term EI even for their models (Bar-On, 1997, 2004; Petrides and Furnham, 2000, 2001, 2003; Petrides et al., 2007a, 2016).

## The validity issue of EI competing measures

The differences among EI models regard also the relative measurement tools and methods. According to Mayer and Salovey (1997) and Mayer et al. (2016), EI should be measured using maximum performance tests, which are usually applied in the field of general intelligence. For this reason, Mayer, Salovey, and Caruso developed the performance test called Mayer-Salovey-Caruso Emotional Intelligence Test for adults (MSCEIT; Mayer et al., 2002), which has been followed by the MSCEIT youth research version (MSCEIT-YRV; Mayer et al., 2014). On the contrary, the sustainers of mixed and trait models used self-report methodologies, often used in the field of personality measurement, releasing widely used self-report scales such as the Emotional Quotient Inventory (EQ-I) based on the Bar-On model (1997, 2004) or the Trait Emotional Intelligence Questionnaire (TEIQue; Petrides, 2009) based on Petrides' model (Petrides et al., 2007a, 2016).

These deep differences in both theoretical models and methodological strategies used to measure EI produced important validity problems. Validation studies of EI tools such as MSCEIT (Mayer et al., 2002) or EQ-I (Bar-On, 1997, 2004) proved that both performance tests and self-report scales owned good reliability, construct validity, and predictive validity. Nevertheless, the associations between EI and other crucial aspects of an individual's physical and psychological life varied according to the ability EI vs. trait EI and performance measures vs. self-report (Zeidner et al., 2005; Fiori and Vesely-Maillefer, 2018; Pérez-González et al., 2020). Even in the case of gender differences, many studies demonstrated that women get higher scores than men in MSCEIT (Mayer et al., 2002; D'Amico and Curci, 2010; Joseph and Newman, 2010; Cabello et al., 2016), but this difference is not always found using self-reports (Charbonneau and Nicol, 2002; Joseph and Newman, 2010).

Most of all, the problem with EI assessment tools concerns the convergent/divergent validity, since individuals' scores in performance and self-report measures are often poorly or not at all related to each other (Brackett and Mayer, 2003; O'Connor and Little, 2003; Warwick and Nettelbeck, 2004; Bastian et al., 2005; Brackett et al., 2006).

In the first studies on this issue (i.e., Matthews et al., 2002), some scholars stated that it might depend on the differences between different EI models (ability *vs*. trait or mixed) underlying each tool. This led to the development of a further group of assessment tools, namely, the Schutte Self-report Emotions Intelligence Test (SSEIT; Schutte et al., 1998), the Self-Rated Emotional Intelligence Scale (SREIS, Brackett et al., 2006), and the Emotional Self-Efficacy Scale (ESE; Kirk et al., 2008) that use self-report methodologies but are focused only on the Mayer and Salovey ability model of EI and do not include personality traits or competencies.

However, Brackett and Mayer (2003) found that SSEIT was weakly correlated with MSCEIT and highly correlated with EQ-I. Similarly, Brackett et al. (2006) obtained low correlations among scores of MSCEIT and SREIS. Finally, Kirk et al. (2008) found an acceptable correlation between ESES and MSCEIT, but ESES scores were highly correlated with SSEIT scores (Schutte et al., 1998).

In conclusion, all these results suggested that differences in the predictive validity of performance and self-report measures, as well as in convergent/divergent validity, might not depend on their underlying theoretical model nor the specific test used. Instead, Brackett et al. (2006) claimed that performance-based measures and self-reports were “most likely tapping into different mental processes” (p. 784) and that low correspondence among individuals' scores in performance-based tests and self-report scales may depend on several factors, such as social desirability response (Paulhus, 1991), low emotional awareness, and lack of metacognitive skills (Brackett et al., 2006).

## Toward the concept of meta-emotional intelligence

All these results appeared interesting and convinced D'Amico (2013) about the importance to assess EI both using self-report and performance tests and, also, to examine the discrepancy among self-report and performance measures under the metacognitive perspective (Flavell, 1979).

D'Amico (2013, 2018) claimed that performance tests may be useful for measuring the actual ability in perceiving, using, understanding, and managing emotions and behavior, but self-report scales are important as well. Perceived emotional abilities, even when they do not correspond to actual abilities, may drive individuals' behavior and choices. Moreover, D'Amico (2013, 2018) guessed that the discrepancy between perceived and actual abilities might offer important insight into individuals' levels of meta-emotional intelligence (MEI), i.e., the metacognition about their emotional intelligence.

Metacognition had been defined by Flavell (1979) as the “knowledge about cognitive phenomena” and consisted of metacognitive knowledge, metacognitive experiences, tasks or goals, and strategies. Since this first conceptualization, other models of metacognition had been proposed that focus on the different subprocesses involved in metacognition (Nelson and Narens, 1994; see Drigas and Mitsea, 2021), and an extensive literature (Roebers, 2017; Norman et al., 2019) had shown how metacognition had a very important regulatory function on cognitive processes. The word metacognition had declined in almost all cognitive processes (meta-comprehension, meta-memory, meta-attention, and so on, see Cornoldi, 1995; Padmanabha, 2020).

Much less attention, however, had been paid to the concept of meta-emotion, at least not under the metacognitive perspective. The term meta-emotions had been used by other authors to refer to “thoughts one makes about emotions” (Briñol et al., 2006); “emotions one feels as a result of one's own or others' emotions” (Gottman et al., 1996; Norman and Furnes, 2016); emotional comprehension and perspective taking (Pons and Harris, 2000; Pons et al., 2002); and awareness about one's own emotions (Lane and Schwartz, 1987) and theory of mind (Lane et al., 2010).

On the contrary, in developing the construct of MEI, D'Amico (2013) focused on three specific metacognitive processes, namely, metacognitive knowledge, metacognitive self-evaluation, and metacognitive beliefs. Metacognitive knowledge is generally described as the awareness and knowledge of one's abilities, potential, and limits; it corresponds to “knowing to know” and allows people to make predictions about possible success/failure in certain situations, guiding them to choose paths and interests that are within their reach, avoiding frustrations, and inducing people to use their strategies to cope with cognitive tasks. In the MEI framework, the term meta-emotional knowledge describes the awareness and knowledge of one's emotional abilities in everyday life.

Metacognitive self-evaluation corresponds to the ability to self-assess one's performance in specific tasks. Those who are capable of self-assessing correctly their performance will be able to correct their mistakes, practice what they have failed to master, and, in short, have a better future performance in similar tasks. In the MEI framework, the awareness and knowledge of one's own abilities in specific emotional tasks are called meta-emotional self-evaluation.

D'Amico (2013, 2018) claimed that low meta-emotional knowledge and meta-emotional self-evaluation are important because they may be responsible for discrepancies among self-report and performance measures in the field of EI. In other words, when people are poor in meta-emotional knowledge or meta-emotional self-evaluation, their self-report or self-rating scores may be untrustworthy since they result from distorted perceptions of their own EI abilities in everyday life or specific emotional performance.

On the contrary, both meta-emotional knowledge and meta-emotional self-evaluation are not so simple to develop, because of the inherent difficulty in self-evaluation of one's emotional abilities. In fact, in all the other cognitive domains, individuals may build metacognitive knowledge and metacognitive self-evaluations based on objective experiences of success or failure (i.e., passing math exams, school grades, succeeding in sports or work, etc.). On the contrary, people do not have great opportunities to get objective feedback in the domain of emotions. Emotional problem-solving is difficult and solutions are uncertain: every day we take emotional decisions and every day we wonder if our decisions are the best ones. In contrast, also for the assessment of EI, Mayer et al. (2002) agreed that not even experts on emotions can evaluate with absolute certainty what is right or wrong in the field of emotions, and for this reason, they used a statistical criterion based on “consensus” for defining the correctness of each answer. For these reasons, it can be difficult for individuals, and even more for preadolescents or adolescents, to become aware of their emotional abilities. Moreover, the judgment about own emotional abilities may be influenced by another important metacognitive factor: the beliefs about emotions. In general, we know that the beliefs that people possess regarding certain aspects of human cognition strongly influence their behavior. For example, people who believe that good memory abilities are an innate gift will make no effort to improve them, while those who believe in the possibility of improving their memory abilities through exercise will be more likely to exercise (Irak and Çapan, 2018). In the MEI framework, the term meta-emotional beliefs indicate the individuals' beliefs (or false beliefs) about the nature, controllability, and usefulness of emotions. Meta-emotional beliefs are highly influenced by education and culture; for instance, we know that suppression may be one of the most dangerous ways to regulate emotions (Richards and Gross, 1999; Gross, 2001; Brockman et al., 2017), but it is also one of the most used in many culture and families (McRae, 2016; Tsai and Lu, 2018). If people believe that suppression is the only way to regulate emotions, they will try to suppress emotions. If they succeed in suppressing emotion, they will believe that they can suppress emotional regulation and so on. Thus, meta-emotional beliefs, as well as meta-emotional knowledge and meta-emotional self-evaluation, may significantly influence the emotional life of individuals and their behaviors (D'Amico, 2013, 2018).

Low MEI (i.e., low awareness of one's emotional abilities and false beliefs about emotions) could arise low meta-emotional control. People with low MEI may choose to cope with a situation they are not able to manage or to avoid a situation that they could. On the contrary, a harmonic MEI profile may help people to cope with situations that they perceive as “within their reach” and to move away or to “let go” of a situation that they consider out of their control.

## The assessment of MEI: The IE-ACCME test

The construct of MEI has been operationalized by D'Amico (2013) developing the Intelligenza Emotiva: Abilità, Credenze e Concetto di Sè MetaEmotivo—Emotional Intelligence: Abilities, Beliefs, and Emotional Self-Concept test (IE-ACCME, D'Amico, 2013), a multi-trait and multi-method assessment tool for preadolescents and adolescents aimed at measuring both EI and MEI.

**Figure 1:**
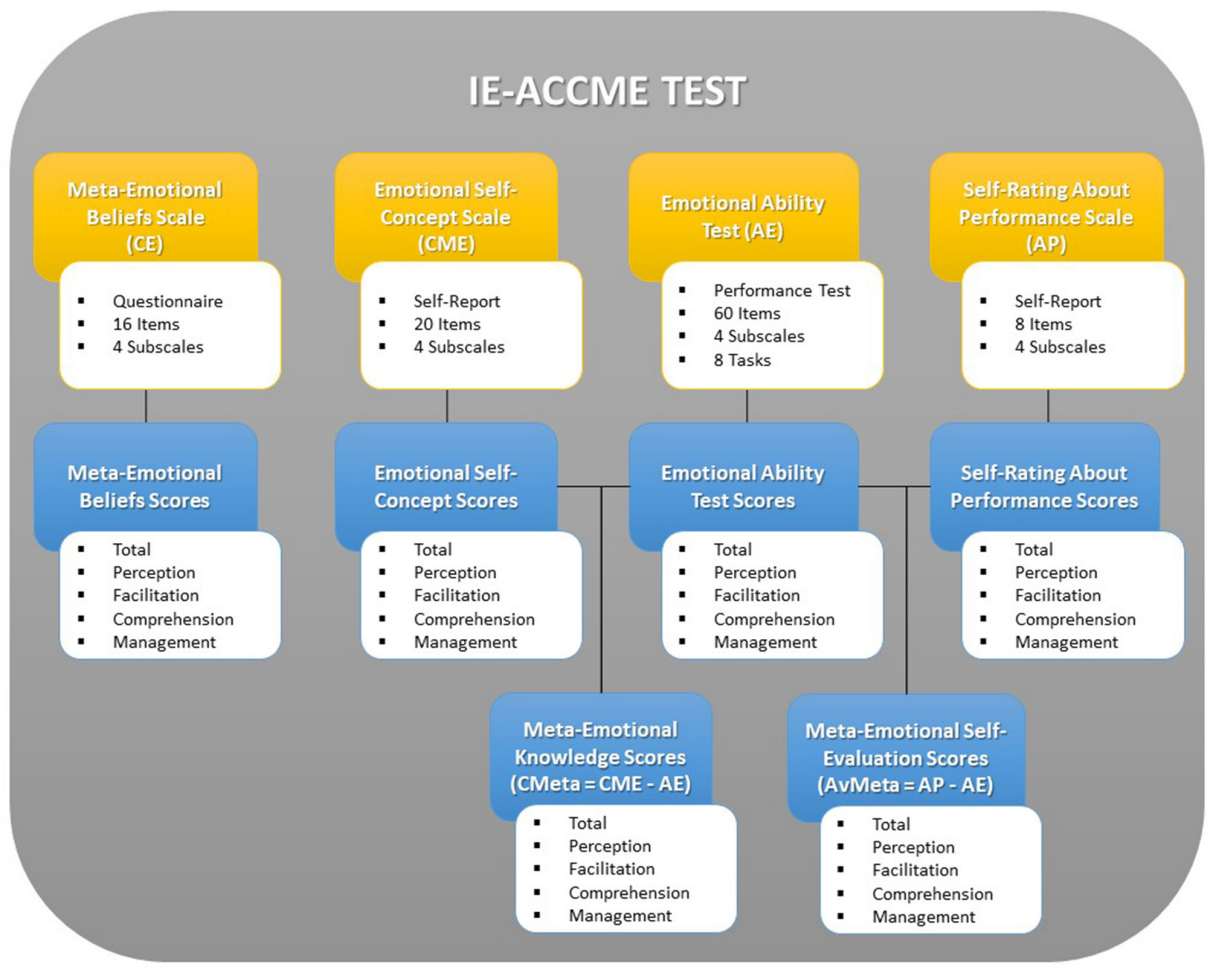
The IE-ACCME test structure. Yellow boxes are the scales and blue boxes are the scores.

The IE-ACCME test (Figure 1) includes four tools given as follows: (1) a questionnaire about meta-emotional beliefs about emotions (CE); (2) a self-report scale on emotional self-concept (CME); (3) a maximum performance test on emotional abilities (AE); and (4) a self-rating scale about one's performance on the emotional abilities test (AP). Moreover, using the score of CME, AE, and AP, the test allows also us to compute the meta-emotional knowledge (CMeta) and meta-emotional self-evaluation scores (AvMeta), which will be described later. All acronyms correspond to Italian subscale names.

Thus, the IE-ACCME test measures different facets of EI using different methods. The only common aspect among IE-ACCME scales is that they all explore the emotional dimensions described in Mayer and Salovey's (1997) four branches of the theoretical model, such as perception of emotions (in faces and pictures), facilitation of emotions in cognitive processes (use and sensations), understanding of emotions (blends and transformations), and management of emotions (personal management and interpersonal management). The IE-ACCME scales are described below:

1. Meta-Emotional Beliefs (CE) is a 16-item questionnaire that explores individuals' beliefs about the role of emotions in everyday life, in perceptions and sensations, and the facilitation of thought, and individuals' beliefs about the possibility of understanding and regulating emotions. Individuals respond on a five-point Likert scale ranging from “not true” to “definitely true”. After validation, however, only eight items, that explained the 60.2% of the variance and focused on the four branches and eight tasks of EI, were selected for computing the CE score. The CE score represents the degree to which people believe that each aspect of emotion included in the EI ability-based model is important and influences everyday life (i.e., if they believe that sensations produce emotions, that emotions can facilitate thinking, that emotions may blend each other, or that emotions can be regulated). Examples of items are: “Only positive emotions help to cope with life” and “In evaluating situations, it is possible to find the best way of behaving with others”. Individuals beliefs are important metacognitive factors since they derive from emotional experiences and influence (and are influenced by) the way we perceive situations, and our choices and preferences.
2. Emotional Self-Concept (CME) is a 20-item scale that explores individuals' perceptions of their emotional skills. Respondents are asked to self-evaluate, rating from 0 to 4 (“not true” to “definitely true”), their ability to recognize emotions in faces, images, and feelings, to use emotions in thought processes, to understand the vocabulary and transformations of emotions, and to manage their emotional states in the personal sphere and relationships with others. Even in this case, the validation procedure revealed that a solution with eight items, focusing on the four branches and eight tasks of EI, explained 60.54% of the variance and was then selected for computing the CME score. Items ask people to evaluate their emotional abilities in everyday situations (e.g., “I can identify the emotions that derive from particular physical sensations”). The self-concept of individuals about their abilities is another important metacognitive dimension since it reflects the way people perceive themselves and their use of emotional strategies in typical situations. Self-concept influences self-confidence in particular domains.
3. The Emotional Abilities Test (AE) is a maximum performance test composed of 8 tasks for a total of 60 items, which measures the emotional abilities of individuals on the perception of emotions (faces and pictures), facilitation of emotions in cognitive processes (use and sensations), understanding of emotions (blends and transformations), and management of emotional problems (personal and interpersonal). The validation procedure revealed that a solution with 54 items, focusing on the four branches and eight tasks of EI, explained 42.12% of the variance and was then selected for computing the scale and subscale scores. The AE scale is inspired by the MSCEIT (Mayer et al., 2002) but its content, the number of items, and the scoring algorithms for each task are different from those used in MSCEIT. Like MSCEIT, the AE scale uses the general and expert consensus methodology so that the score of each answer is proportional to the number of times that it was chosen from people in the general or expert standardization samples. Thus, the best responses are those with the highest frequency in the general or expert standardization samples (D'Amico, 2018). The general standardization sample was composed of 1,084 Italian preadolescents and adolescents (526 male subjects and 558 female subjects; between 10 and 19 years of age; recruited in southern, central, and northern Italy). The expert standardization sample was composed of 40 Italian scholars of emotions (11 men and 29 women; academics, clinicians, graduate students, and interns; recruited in southern, central, and northern Italy). Results demonstrated that there is a high correlation (r = 0.64, *p* < 0.001) among total AE scores computed using the general and expect consensus score (D'Amico, 2013).
4. Self-Rating of Performance (AP) is an 8-item scale distributed throughout the emotional ability test. At the end of each task, the respondent is asked to self-evaluate his/her performance, choosing a score from a six-point Likert scale from “not good at all” to “very good”. A comparison between performance in the ability test (AE scores) and self-evaluation of performance (AP scores) may give important insights into individuals' metacognitive awareness. As many studies on metacognition have stated, self-assessment is an important process to know the degree of understanding and attention that individuals lend to a particular task, the degree of the perceived difficulty of the task, and the degree of awareness of their performance in the same task.

## Psychometric properties of the IE-ACCME test scales

The IE-ACCME test has undergone a validation and standardization process aimed to examine the psychometric properties of IE-ACCME scales and in particular (1) apparent validity; (2) structural validity; (3) scale intercorrelations; (4) reliability and test-retest stability; (5) convergent and discriminant validity about classical measures of verbal and non-verbal intelligence, other measures of EI and scales of personality; and (6) predictive discriminant validity about school achievement (for further details, see D'Amico, 2013);

Validation and standardization involved the general sample described before and composed of 1,084 participants (526 male subjects and 558 female subjects; between 10 and 19 years of age; recruited in southern, central, and northern Italy).

In developing each IE-ACCME scale, a deep analysis of EI theoretical models and the relative measurement tools was conducted. In this sense, the apparent validity of the test is quite strong, and people in the normative sample recognize it as a tool intended to measure emotions.

Structural validation was performed using explorative and confirmatory factorial analyses and demonstrated that IE-ACCME scales (CE, CME, and AE) reflect Mayer and Salovey's (1997) four branches and eight tasks structural model. In the case of the AE scale, the model was confirmed both when scores were computed using the general and the expert consensus sample. However, analyses of scale intercorrelations demonstrated that scores of CE, CME, AE, and AP are very slightly correlated or not correlated with each other (AE vs. CME: *r* = 0.04, *p* > 0.05; AE vs. AP: *r* = 0.09, *p* < 0.01). A significant correlation was found only between AE and CE (*r* = 0.31, *p* < 0.05), indicating that people with a belief system about emotions, that is consistent with EI theorization, own also good levels of emotional abilities and vice versa. CE total score is only slightly related to CME total score (*r* = 0.18, *p* < 0.01) and the AP total score (*r* = 0.14, *p* < 0.01); similarly, CME total score is slightly related to the AP total score (*r* = 0.179, *p* < 0.01). These results confirm that each IE-ACCME scale not only uses different methods but also measures distinct aspects of the emotional sphere.

Concerning reliability, Cronbach's alpha was not computed for the IE-ACCME total scores, due to the small number of items in the CE, CME, and AP scales (8), and because the items in the total AE scale are heterogeneous (D'Amico, 2013). However, the AE scale presented a good split-half value (*r* = 0.86), and test-retest stability, explored in a subgroup of 96 adolescents, was acceptable considering the small sample size (CE, *r* = 0.43, *p* < 0.001; CME, *r* = 0.76, *p* < 0.001; AE, *r* = 0.44, *p* < 0.001; AP, *r* = 0.55, *p* < 0.001).

Convergent and discriminant validity of the IE-ACCME test with regard to other aspects of intelligence was performed in a subgroup of 388 participants (173 male subjects and 215 female subjects) drawn from the general normative sample. Participants' IE-ACCME scores were compared to participants' scores in tests of non-verbal and verbal abilities, respectively, assessed using standard progressive matrices (SPM; Raven and Raven, 2003) and the verbal meaning subtest from the PMA battery (PMA-VM, Thurstone and Thurstone, 1965). Results demonstrated that some CE scores showed convergent validity about the measure of nonverbal ability, while the CME scale showed neither convergent nor discriminant validity about either verbal or nonverbal ability. AE scale, finally, showed a good degree of both convergent and discriminant validity about both verbal and nonverbal ability. However, none of the significant correlations obtained among IE-ACCME scores and verbal and nonverbal ability scores exceeded *r* = 0.26, demonstrating that there is no overlapping among the psychological dimensions measured in the different tests.

Convergent and discriminant validity of the IE-ACCME scales (CE, CME, and AE) toward other EI tests and personality scales was assessed in a small subgroup of 96 participants drawn from the general normative sample to complete also the Italian version of MSCEIT (Italian version by Mayer et al., 2002; D'Amico and Curci, 2010), the EQ-I (Bar-On, 1997; Italian version by Franco and Tappatà, 2009), and the BFA scale for the assessment of big five factors of personality (Caprara et al., 1993). D'Amico (2013) expected to find a convergence between participants' scores on the AE scale and MSCEIT since they are based on the same theoretical model and are both performance tests. On the contrary, low or no correlation was expected between CE and CME scores and MSCEIT, since they use different measurement methods (questionnaire, self-report, and performance tasks), even measuring the same theoretical variables. D'Amico (2013) expected to find no correlation between none of IE-ACCME scores and EQ-I scores since they are based on different theoretical models of EI and use different measurement methods (Brackett and Mayer, 2003; O'Connor and Little, 2003; Warwick and Nettelbeck, 2004; Bastian et al., 2005; Brackett et al., 2006). Concerning the associations between IE-ACCME scales and BFA scale, D'Amico (2013) expected to find only some correlation between CME and BFA emotional-related variables (Davies et al., 1998; Dawda and Hart, 2000; Matthews et al., 2004; van der Linden et al., 2017; Bucich and MacCann, 2019) since they are both based on self-report, whereas non-association was expected between AE and BFA since they reflect two different psychological dimensions (beliefs, EI abilities, and personality dimensions) and use different measurement method (questionnaire, performance tasks, and self-report).

Even if they are based on a small sample of participants, results confirmed the expectations, demonstrating that there were neither significant correlations between CE and CME scores and MSCEIT nor none of the IE-ACCME scores and the EQ-I scores. On the contrary, there was a significant correlation between some of the AE branch or task scores and some of the MSCEIT branch or task scores (i.e., the AE Facilitation branch score was associated with the MSCEIT management branch score, and the AE blending task score was associated with MSCEIT perception and management branch scores), even if there were no association between AE and MSCEIT total score. In this regard, it is important to stress that AE and MSCEIT tests are addressed to populations of different ages (10–18 years vs. > 18 years), and, consequently, they use items of quite different difficulty levels, and situations and scenarios used in subtests are vastly different.

Concerning the relationship between IE-ACCME scores and personality factors, an association was found between CME and agreeableness, whereas no association was found between CE total score and none of the personality factors. Surprisingly, there were many correlations between AE scores and personality factors: total AE score was associated with energy, agreeableness, and openness. The range of correlation did not exceed *r* = 0.40 (between total AE score and openness), which is high enough to demonstrate that the two dimensions are associated, but not so high demonstrating that there is an overlapping between the AE scale and personality factors.

Finally, studies performed during IE-ACCME psychometric test validation demonstrated good predictive and discriminant validity of the IE-ACCME test about school achievement. School achievement of a subgroup of 133 participants drawn from the total sample was measured using teachers' evaluations 1 year after the test administration. Correlational analyses between IE-ACCME scores and teachers' evaluations demonstrated that CE and CME scores were not related to teachers' evaluations, whereas there was a predictive association between some AE scores (branches perception and management) and teachers' evaluations.

Thus, consistent with previous literature (Lyons and Schneider, 2005; Gil-Olarte Márquez et al., 2006; Mestre et al., 2006), EI is predictive of school achievement mostly when it is measured as an array of abilities using a performance-based test, and not always when it is measured using self-report scales (Newsome et al., 2000; Van Der Zee et al., 2002; Barchard, 2003; Bastian et al., 2005).

## MEI profiles and MEI composite scores

As mentioned above, the results of psychometric validation of IE-ACCME test scales proved that all of them reflect Mayer and Salovey's (1997) four branches and eight tasks structural model. However, scores of CE, CME, AE, and AP are not or very slightly correlated with each other, indicating that they measure different processes of the emotional sphere.

**Figure 2:**
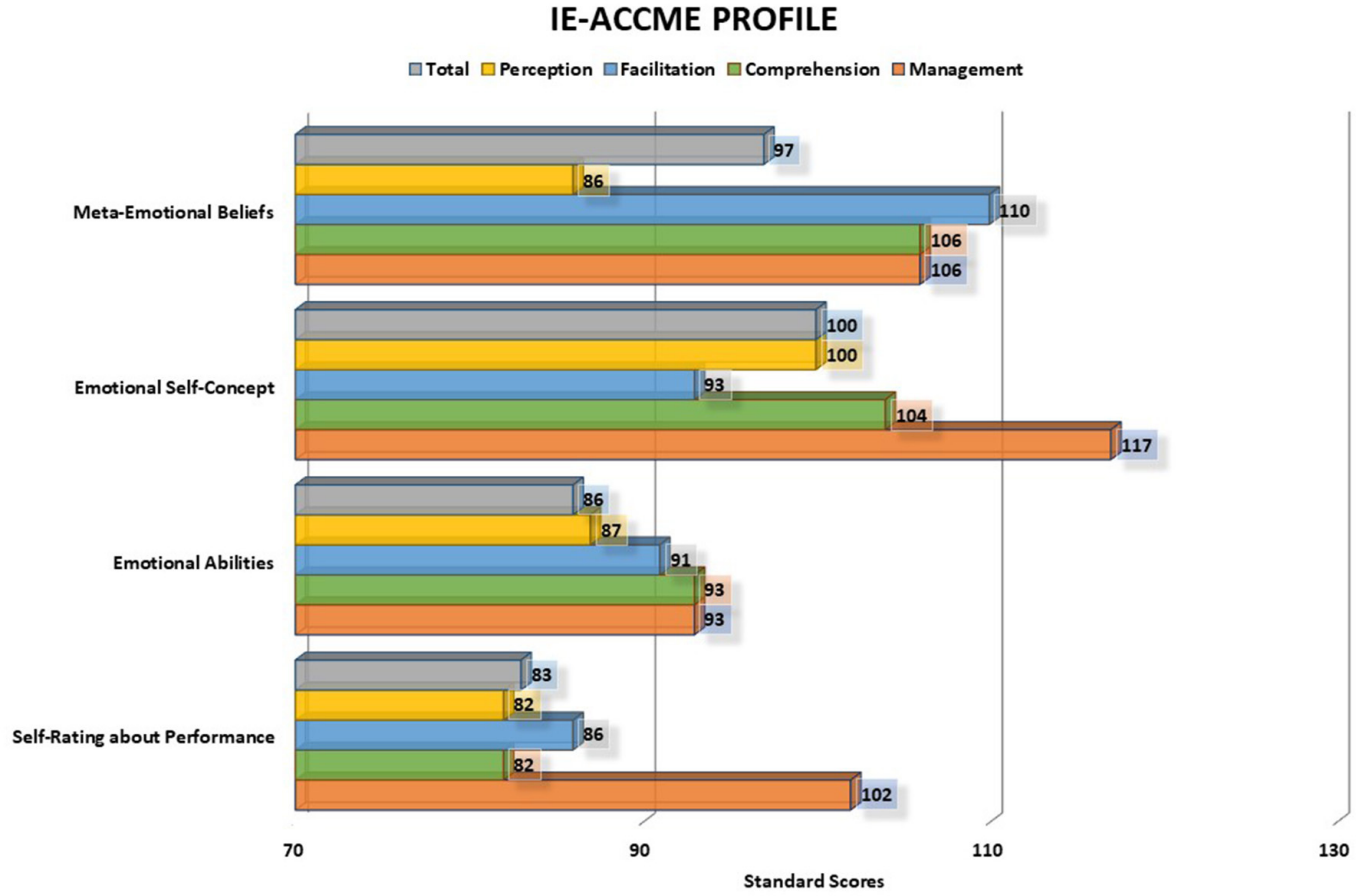
IE-ACCME profile of an adolescent boy. For each scale, the chart presents the total score and the score in the perception, facilitation, understanding, and management branches. In the example, there are differences in scores between the scales (the scores in the emotional ability test and self-evaluation of performance are generally lower than others) and there are also differences among the four branches of EI in each scale.

From the different IE-ACCME scores, it is possible to build both EI and MEI profiles of each teenager and highlight strengths and weaknesses in each scale and subscale. Each total scale and subscale score of CE, CME, AE, and AP are expressed as standardized scores with a mean of 100 and a standard deviation of 15, and this allows for comparing the scores obtained in different subtests. Thus, for instance, focusing on the emotional ability test (AE), it is possible to evidence if the ability of emotional perception is higher than the ability to manage emotions (see possible profile in Figure 2).

Moreover, by comparing the scores of different subscales, it is possible to examine their discrepancies and compute the meta-emotional knowledge and meta-emotional self-evaluation scores:

A. Meta-emotional knowledge score (CMeta) corresponds to the discrepancy between scores obtained on the emotional ability test (AE) and emotional self-concept scale (CME) and indicates the extent to which the subject's performance on the ability test corresponds to the perceptions of ability in everyday life. A discrepancy score higher than 15 standardized points is considered to indicate a low meta-emotional knowledge. Scores may fall in the area of overestimation (positive scores) or underestimation (negative scores): low (below ±15), medium (between ±15 and ±30), and high (between ±30 and ±45).
B. Meta-emotional self-evaluation score (AvMeta) corresponds to the discrepancy between the scores obtained in the emotional ability test (AE) and the self-rating about performance scale (AP) indicating the extent to which the subject's performance on the ability test corresponds to self-assessments of performance on the test. A discrepancy score higher than 15 standardized points is considered to indicate a low meta-emotional self-evaluation. Scores may fall in the area of overestimation (positive scores) or underestimation (negative scores): low (below ±15), medium (between ±15 and ±30), and high (between ±30 and ±45).

**Figure 3:**
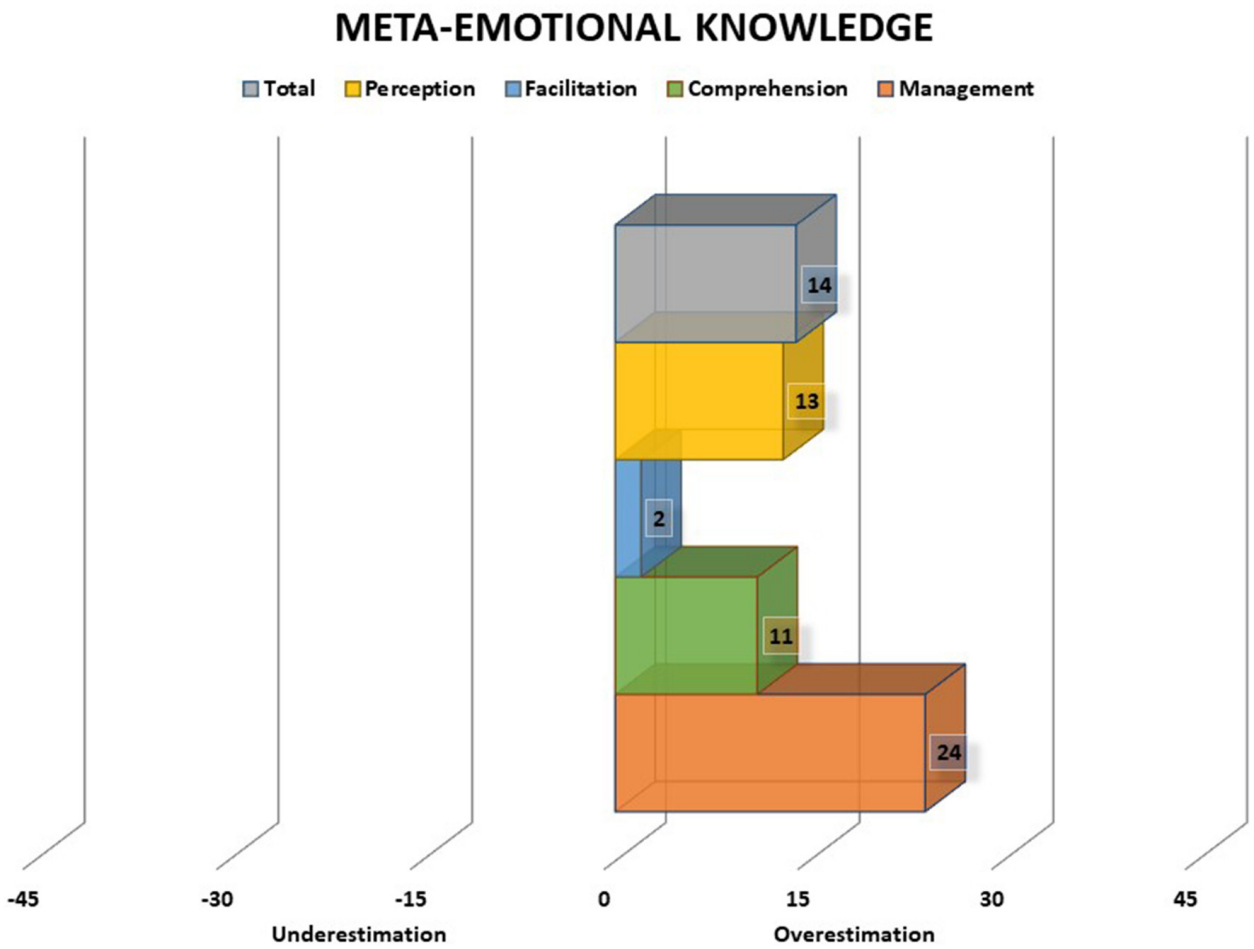
Meta-emotional knowledge scores of an adolescent boy. The chart presents total score, perception, facilitation, understanding, and management scores. Scores may fall in the area of overestimation (positive scores) or underestimation (negative scores): low (below ±15), medium (between ±15 and ±30), and high (between ±30 and ±45). In the example, there is a general tendency to overestimation, which reach the medium level in the management branch. Thus, in general, the individual's self-perception of emotional abilities in everyday life is higher than his actual performance on the test.

**Figure 4:**
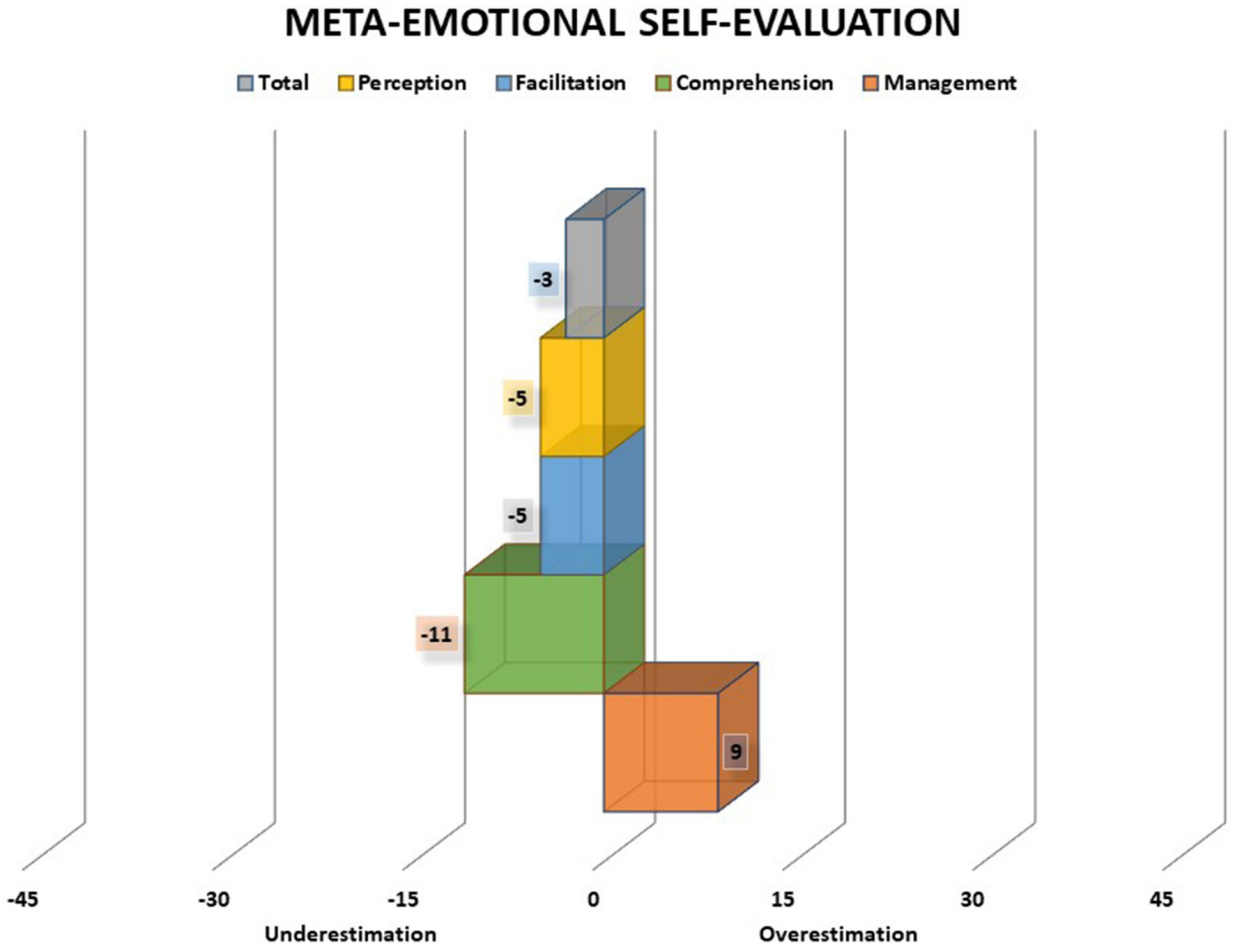
Meta-emotional self-evaluation scores of an adolescent boy. The chart presents total score, perception, facilitation, understanding, and management scores. Scores may fall in the area of overestimation (positive scores) or underestimation (negative scores): low (below ±15), medium (between ±15 and ±30), and high (between ±30 and ±45). In the example, there is a general tendency to underestimation, except for the overestimation score in the management branch. However, all discrepancy scores are low and none of them reach the level of one standard deviation (15 standardized points). Thus, in general, there is an acceptable correspondence between the performance on the ability test and the self-perception of performance.

Both meta-emotional knowledge and meta-emotional self-evaluation scores may present positive or negative values. In this sense, positive values indicate an overestimation of one's emotional abilities in daily life and/or in testing situations, whereas negative values indicate an underestimation of one's emotional abilities in daily life and/or in testing situations (see examples in Figures 3, 4).

A harmonious MEI profile occurs when all scores in the ability test, as well as in the meta-emotional belief, emotional self-concept, and self-rating about performance, are quite similar and do not show variations that overcome one standard deviation (i.e., 15 standardized points). The optimum is represented by individuals who obtain a harmonious MEI profile that is accompanied by high scores in the ability test, as well as in the meta-emotional belief, emotional self-concept, and self-rating about performance scales.

Low MEI profiles, on the contrary, occur in case of higher scores of false beliefs, or underestimation or overestimation of one's emotional abilities. In case of underestimation, a teenager may show a high score on the emotional abilities test and a low score on the emotional self-concept or self-assessment. In case of overestimation, a teenager may show a low score on the emotional abilities test and a high score on the emotional self-concept or self-assessment. As claimed above, from the author's perspective (D'Amico, 2018), poor MEI, both when it depends on false beliefs or underestimation and overestimation, may be dangerous for emotional life: false beliefs may influence the way people live their emotional experiences; overestimation of one's emotional abilities might lead adolescents to cope with situations they are not able to manage; underestimation of their emotional abilities might lead them to avoid those situations that they could be able to front, reducing the experiences of success.

Recent studies also allowed us to study in more detail the predictive validity of MEI scores toward sociometric status (D'Amico and Geraci, 2021) and for explaining sex differences (D'Amico and Geraci, 2022a). The first study (D'Amico and Geraci, 2021) involved a sample of 105 students (55 female subjects and 50 male subjects), between 10 and 16 years (M = 12 years and 6 months; SD = 15.27 months) from lower secondary schools. The sociometric status was assessed through a sociogram and by asking classmates to choose, those whom they would/would not like to make schoolwork with. EI and MEI were measured using the IE-ACCME test. Since in this case, it was necessary to compare group MEI scores of preadolescents and adolescents, the discrepancies between CME, AP, and AE were weighed on the AE score to control individual differences in ability EI (thus, the MEI weighed score were computed as follows: CMeta = CME–AE/AE; AvMeta = AP–AE/AE).

Our initial hypotheses were that students who possess higher emotional abilities tend to be more accepted and less rejected by their classmates compared to those students who possess lower emotional abilities. As for the relationships among meta-emotional dimensions, wellbeing, and sociometric status, there were no previous studies about this issue. However, we hypothesized that those preadolescents with higher levels of meta-emotional knowledge and/or meta-emotional self-evaluation, being more aware of their emotional abilities, could experience higher wellbeing and own higher sociometric status than others. For preadolescents with poor meta-emotional knowledge and/or poor ability in meta-emotional self-evaluation, we were also very curious to know which type of esteem error (overestimation vs. underestimation) could negatively influence eudemonic wellbeing and sociometric status.

Finally, we hypothesized that owning meta-emotional beliefs that are consistent with what current theories and empirical evidence about EI demonstrated could be positively associated with emotional ability, eudemonic wellbeing, and sociometric status. As expected, results showed a positive relationship between emotional abilities and social status. New and interesting information stems from MEI results since they demonstrated that those who possess adequate meta-emotional knowledge are more accepted by their peers compared to the overestimating classmates who tend to be more rejected by peers. Similarly, adolescents who presented accurate meta-emotional self-evaluation showed higher rates of acceptance and lower rates of rejection by peers. In addition, we found that preadolescents' psychological wellbeing was predicted by their meta-emotional beliefs. These results shed new light on the relationship between emotional abilities and adolescents' social success. For young people to engage in functional social relationships, being highly emotionally intelligent is not the sole condition: they need to be aware of their abilities. Those who overestimate their emotional abilities are more rejected compared to those who underestimate them, probably because they tend to engage in situations that they cannot manage. On the contrary, underestimation might have its downsides since it might lead adolescents to avoid those situations that they might be able to manage, experiencing less emotional efficacy. Arguably, compared to overestimating people, those who underestimate are less rejected because they are less “seen” by others.

The second study allowed us to examine the validity of IE-ACCME scores in discriminating among sexes (D'Amico and Geraci, 2022a). As already demonstrated in the IE-ACCME test validation (D'Amico, 2013) and consistently with scientific research (Mayer et al., 2002; Day and Carroll, 2004; D'Amico and Curci, 2010; Fernández-Berrocal et al., 2012; Rivers et al., 2012; Cabello et al., 2016; Gutiérrez-Cobo et al., 2016), there are important sex differences in EI across the lifespan. However, there are some inconsistencies in the results from the literature: both women and girls score higher than men and boys in performance tests (Mayer et al., 2002; Brackett and Mayer, 2003; Day and Carroll, 2004; D'Amico and Curci, 2010; Fernández-Berrocal et al., 2012; Cabello et al., 2016; Gutiérrez-Cobo et al., 2016), whereas, in self-report scales, women/girls score higher than men/boys only in some subscale and sometimes male subjects score higher than female subjects (Dawda and Hart, 2000; Petrides and Furnham, 2000; Ciarrochi et al., 2001; Brackett and Mayer, 2003; Meshkat and Nejati, 2017; D'Amico et al., 2020). Thus, the size and directions of sex difference in EI depend on the type of measurement tool used (ability test vs. self-report scale). In our study, we hypothesized that these results may also depend on low individuals' MEI. We evaluated this hypothesis in a study involving 519 preadolescents and adolescents (295 girls and 224 boys). As expected, results showed that girls performed better than boys in the emotional abilities test, whereas boys report higher self-report EI than girls. Regarding meta-emotional knowledge, in the preadolescent group, both boys and girls slightly overestimated their emotional abilities, but in the adolescents' group, boys tended to overestimate their emotional abilities in everyday life compared to girls who, on the contrary, underestimated their emotional abilities. The same pattern was found for meta-emotional self-evaluation, since, in between the preadolescent and adolescent groups, boys tended to overestimate their performance in the ability test instead of girls who tended to underestimate it. Thus, opposite overestimation and underestimation tendencies in the two sexes amplify the distances between the emotional world of boys and girls, increasing the gender gap. In addition, we found that girls possess higher levels of meta-emotional beliefs than boys, and this tendency remains stable over time.

## The utility of the MEI framework

The results above described provided evidence for the utility of the new MEI framework for understanding inter-individual and intra-individual differences.

Another fundamental aspect of MEI is related to its higher plasticity compared to EI, as demonstrated in our recent study on the first application study of MetaEmotions at School (D'Amico and Geraci, 2022b), a program for improving meta-emotional intelligence in the school context. A sample of 264 pupils (M = 129, F = 135; average age = 11.95, SD = 0.27), from lower secondary schools, participated in a quasi-experimental two-group pretest and posttest research design. Once again, both EI and MEI scores were computed by weighing emotional abilities scores for controlling individual EI differences. Results showed that emotional ability scores in the intervention groups tended to be stable over time when compared to the comparison groups. Thus, the program had no effect on improving EI in pupils. However, participants in the intervention groups showed a reduced tendency than participants in the comparison group in overestimating their emotional abilities. In addition, participants in the intervention group showed an increase in their levels of meta-emotional beliefs compared to comparison group ones. This evidence suggests that if emotional abilities reflect a form of intelligence and, as such, require time to be improved and sometimes do not show important variations (Nelis et al., 2009; Dacre Pool and Qualter, 2012), MEI is a malleable dimension since people may modify their beliefs and convictions about emotions and may become more aware of their emotional abilities in a brief period if well guided.

Developing a belief system that reflects actual scientific knowledge about emotions and becoming more aware of one's emotional abilities may have important effects on self-regulation and in general on individual life. As already claimed, emotional abilities may enable people to perceive, use, understand, and manage emotions and actions, but meta-emotional dimensions drive individuals' choices and behaviors (D'Amico, 2013, 2018).

## Conclusion and future directions

Meta-emotional intelligence is a new construct that may provide new and innovative insights into EI research. It represents a comprehensive theoretical framework that brings together different EI measurement methods and explains the existing gap between measure outcomes.

Although the study of MEI is newly born, the tool for the assessment of emotional and meta-emotional profiles in preadolescents and adolescents is available, and as already claimed by D'Amico and Geraci (2022a), it may give important insights to professionals for (a) promoting the awareness of preadolescents and adolescents' emotional abilities to reduce the possible overestimation and underestimation bias in evaluating their emotional abilities; (b) stimulating, particularly in male subjects, their habits to share emotions with others and to be attentive about their feelings; (c) discussing with preadolescents and adolescents their meta-emotional beliefs and the cultural misconceptions about emotions.

Current research efforts on MEI are also focused on the development of two assessment tools, respectively, designed for children and adults. We are convinced that a complete assessment of MEI should precede any program for empowering emotional intelligence in children, adolescents, and adults. In fact, to stimulate meta-emotional awareness of one's strengths and areas for improvement and to maximize the effects of training, the latest version of the MetaEmotions program involves, as the first step of the program, the assessment of MEI and the discussion of each participant's results.

## Author contributions

AD'A conceived the theoretical framework of the article. AD'A and AG designed, reviewed, and revised the manuscript and approved the final manuscript. All authors contributed to the article and approved the submitted version.
